# Barrier or stressor? The role of discrimination experiences in health service use

**DOI:** 10.1186/s12889-018-6267-y

**Published:** 2018-12-07

**Authors:** Billy Gazard, Zoe Chui, Lisa Harber-Aschan, Shirlee MacCrimmon, Ioannis Bakolis, Katharine Rimes, Matthew Hotopf, Stephani L. Hatch

**Affiliations:** 1 Psychological Medicine, Psychology and Neuroscience, King’s College London, Institute of Psychiatry, London, UK; 2 Department of Public Health Sciences, Karolinska Institutet, Stockholm, Sweden; 3 Centre for Implementation Science, Health Services and Population Research Department, Psychology and Neuroscience, Institute of Psychiatry, King’s College London, London, UK; 4 Department of Biostatistics and Health Informatics, Psychology and Neuroscience, Institute of Psychiatry, King’s College London, London, UK; 5 Psychology and Neuroscience, King’s College London, Psychology, Institute of Psychiatry, London, UK; 6 South London and Maudsley NHS Foundation Trust, London, UK

**Keywords:** Epidemiology, Health service use, Discrimination, Population survey, Intersectional approaches, Community health

## Abstract

**Background:**

Discrimination is a well-established stressor that is substantially associated with poor health and a known contributor to health inequalities. However, the role of discrimination in health service use is less explored. This study will take an intersectional approach to investigate differences in health service use and examine the role of discrimination experiences.

**Methods:**

Data on health service use were assessed in a diverse inner London sample of 1052 participants in the South East London Community Health (SELCoH) Study. Latent class analysis (LCA) was used to define classes of intersectional social status using multiple indicators of socioeconomic status (SES), ethnicity and migration status. Adjusted associations between intersectional social status and discrimination experiences with health service use indicators are presented.

**Results:**

Using latent class analysis allowed us to identify an intersectional social status characterized by multiple disadvantage that was associated with decreased secondary physical health service use and a class characterized by both privilege and disadvantage that was associated with increased health service use for mental disorder after controlling for age, gender and health status. Anticipated discrimination was also associated with increased service use for mental disorder in adjusted models. There was no evidence to suggest that discrimination experiences were acting as a barrier to health service use.

**Conclusions:**

This study highlights the complex ways in which discrimination experiences may increase the need for health services whilst also highlighting differences in health service use at the intersection of ethnicity, migration status and SES. Findings from this study illustrate the importance of measuring multiple levels of discrimination and taking an intersectional approach for health service use research.

## Background

Discrimination is a well-established stressor that substantially contributes to common mental disorders (CMD) and poor physical health [1–3]. Recent research in the UK has found the effects of discrimination on CMD to be more pronounced for ethnic minority groups and those who had recently migrated to the UK, and that cumulative exposure to discrimination experiences has incremental negative long term impacts on mental disorder [4, 5]. Although less explored, discrimination within healthcare settings is also widely acknowledged. Such experiences are generally conceptualized as barriers to utilization, and a recent systematic review reported perceived discrimination to be associated with both negative patient experience and avoidance of healthcare services [6]. However, research has primarily focused on the impact of racial discrimination and racial biases among healthcare practitioners [7–11]. Less attention has been paid to the contribution of discrimination related to other social statuses and the cumulative impact of discrimination experienced across multiple life domains.

Discrimination experiences elicit a variety of psychological and behavioural responses that can impact on individual’s health and health service use [12]. In health service use contexts, mistrust of mental health services has been shown to mediate the association between perceived discrimination and poor health service engagement [13, 14]. Such responses to discrimination experienced in healthcare settings or across other life domains can lead to anticipated discrimination and avoidance of services [15, 16]. In the UK, where secondary health services include planned hospital care, such as inpatient stays or outpatient appointments for treatment or check-ups, both experienced and anticipated discrimination are likely to play an important role in service use as patients are often required to negotiate access with healthcare providers [17]. This may provide an explanation for the observed decrease in secondary health service use for marginalized groups e.g. ethnic minorities, migrants and low SES groups, after accounting for need (e.g. health status) in universal healthcare contexts across the UK and Europe more widely [18–22].

### Importance of taking an intersectional approach

Intersectionality proposes examination of multiple aspects of identity simultaneously to determine how privilege and disadvantage surrounding individuals’ identities interlock [23]. Although intersectional approaches have predominantly been used in qualitative research, it has also been used in quantitative research as a framework for data reduction to understand health differences at the intersection of multiple social statuses in diverse populations. In utilizing approaches such as stratification and latent class analysis (LCA) to derive classes of intersectional social status, we have found health inequalities at the intersection of ethnicity, migration status and SES [24, 25] that were not identified when considering single indicators alone [26]. Both discrimination experiences and access to health services are socially distributed, with higher prevalence of perceived discrimination and lower secondary health service use for ethnic minority, migrant and low SES groups [4, 27, 28]. Taking an intersectional approach allows for the exploration of how these social statuses interrelate and the identification of how discrimination experiences influence health service use at specific intersections of social identity.

Thus, the aims of this study are to (1) determine the distribution of discrimination by intersecting social statuses; (2) identify differences in health service use after accounting for health status (mental/physical disorder and long-standing illness) and (3) examine the role of discrimination experiences in identified differences in health service use after accounting for intersecting statuses and health status. We hypothesized that those with multiple disadvantaged social statuses will use less health services than those with privileged or singularly disadvantaged social status after controlling for health status. We also hypothesized that discrimination experiences will act as a barrier to health service use such that adjusting for these experiences will attenuate associations between disadvantaged social status and decreased health service use.

## Methods

### Sample and procedure

The South East London Community Health (SELCoH) study is a UK psychiatric and physical morbidity survey of randomly selected households from two boroughs in South East London, Lambeth and Southwark. The survey assesses demographic and socio-economic characteristics; physical health and mental disorder; treatment and service use; and social adversity [4, 26]. Households were identified through stratified random sampling, applying similar methods to those of the National Psychiatric Morbidity Surveys. This involved randomly sampling addresses from the Small User Postcode Address File, which excludes addresses receiving more than 50 items of post per day. Letters describing the study were sent to all private households inviting those aged over 16 years old to participate. Detailed information about the recruitment procedures has previously been reported [26].

SELCoH 1 (2008–2010) included 1698 adults from 1075 households (household response rate: 51.9%, within-household participation rate: 71.9%) [26]. SELCoH 2 (2011–2013) targeted 1596 participants who agreed to be re-contacted. 1052 participants were interviewed (response rate: 73%) using a computer assisted interview schedule; 1022 were face-to-face interviews in their households and 30 (2.9%) interviews were conducted using Computer Assisted Telephone Interviews to access participants who were temporarily located outside of London during the data collection [4]. Unless otherwise stated the current analyses refer to data from SELCoH 2.

### Measures

#### Health service use

For health service use relating to mental disorder, participants were asked if they had spoken to a General Practitioner (GP), mental health specialist, psychological therapist or counsellor in the last 12 months about problems with emotions or nerves or their use of alcohol or drugs. For secondary physical health service use, we asked participants if they had been to a hospital for treatment or check-ups in the last 12 months for physical health problems.

#### Discrimination

Everyday discrimination was evaluated with ten items that asked respondents about the occurrence of discrimination experiences that may happen in their day-to-day life (e.g. being called names or insulted) [Everyday Discrimination Scale; 30]. Responses to these 10 items were dichotomised into often/sometimes (1) versus almost never/never (0). Items were summed to a total score and a dichotomous variable was created with 1 if the total score for each participant was above the median and 0 if the total score was below (range 0–10; median 1.0; weighted mean 2.04). Those participants who reported everyday discrimination were also asked for the perceived main reason for these experiences; race/ethnicity; national origins; education or income; age; gender; weight; sexual orientation; religion; physical disability; mental illness; appearance; and other. Due to small cell sizes, weight, sexual orientation, religion, physical disability, mental illness and appearance were collapsed and incorporated into the Other category. Major experiences of discrimination were measured by asking participants if they have ever (yes/no) been unfairly treated across different life domains, such as employment, housing and healthcare, as outlined in previous studies [4, 29]. This measure was similar to Williams et al. [30] with the exception of two additional domains, the court system and public transport. Items were summed and a three-category variable was created around the median and ninetieth percentile of events experienced to improve distribution (range 0–9; median 1.0; weighted mean 1.04). Items measuring anticipated discrimination were taken from the Discrimination and Stigma scale (DISC) [15] and were modified to capture the extent to which participants had stopped themselves from applying for work or for training/education; contacting health services; and going into certain areas/neighbourhoods. For the current study, responses were dichotomised into a little/somewhat/a lot versus not at all after detecting evidence of skewness with the distribution. Items were summed and a categorical variable was created (none, one domain, two or more domains).

#### Sociodemographic and socioeconomic indicators

Participants were asked to self-identify their ethnicity according to UK census categories. Ethnicity categories were collapsed into the following categories; White British, Black Caribbean, Black African, White Other, Non-White Other and Mixed ethnicity. The White Other ethnic group primarily included participants from North Africa and other European countries; the Non-White Other group included Indian, Pakistani, Chinese, Latin American and other Black and Asian groups. Migration status was captured by asking participants their country of birth and length of stay in the UK to create four migration status categories; born in the UK, migrant 0–10 years, migrant 11–20 years, and migrant 21 or more years. Socioeconomic indicators for LCA included educational attainment, social occupational class (SOC) [31], employment status, household income, benefit receipt, debt, tenure and residential mobility. More detailed information on how these indicators were measured and entered into LCA models can be found in a previously published study [25]. Age and gender were also used for descriptive analyses and for inclusion as potential confounders in logistic regression models.

#### Health status

Symptoms of common mental disorders (CMD) were assessed by the Revised Clinical Interview Schedule (CIS-R), a structured interview that asks about 14 symptom domains: fatigue, sleep problems, irritability, worry, depression, depressive ideas, anxiety, obsessions, subjective memory and concentration, somatic symptoms, compulsions, phobias, physical health worries and panic [32]. Participants were classified as having CMD if they scored 12 or more on the CIS-R. Physical disorder was assessed by the Patient Health Questionnaire (PHQ-15) that assesses 15 somatic symptoms [33]. A total score of 10 or more was used to indicate the presence of moderate somatic symptoms [33]. Participants were classified as having a long-standing illness if they reported having a long-standing illness, such as depression, diabetes or asthma.

#### Life events

Life events were assessed using 20 questions that asked about the experience of stressful events over the lifetime and were selected from checklist measurements from the literature on stressful experiences relevant to diverse inner city populations, such as experience of childhood sexual abuse, having a serious accident and witnessing violence or murder [34, 35]. Response categories were binary (yes/no). A three-category variable was created around the median and ninetieth percentile of events experienced to improve distribution (range 0–14; median 4.0; weighted mean 4.1).

#### Latent class analysis

LCA was conducted to define groups with similar social status profiles based on the above measures of SES, ethnicity and migration status. LCA is an established data-driven statistical method which classifies individuals in a sample based on conditional probabilities [36]. Individuals in each assigned class will have a similar pattern of responses based on variables entered into the model. All analyses were conducted in MPlus 6 [37] and followed the same methodology to define latent classes of social status as previously reported in this sample [25]. The combination of these social indicators in LCA analysis produced 7 classes of intersectional social status that represented privileged, mixed and disadvantaged positions, reflective of the study sample; (1) ‘Migrant, mixed ethnicity, low SES’ (*n* = 100), (2) ‘White British, low SES’ (*n* = 107), (3) ‘Non migrant, mixed ethnicity, student’ (*n* = 106), (4) ‘Non migrant, mixed ethnicity, skilled’ (*n* = 153), (5) ‘Mixed migration status, mixed ethnicity, economically inactive’ (n = 100), (6) ‘Migrant, mixed ethnicity, high SES’ (*n* = 181) and (7) ‘White British, high SES’ (*n* = 305) (Table 1). Detailed information of these latent classes are available elsewhere [25].

**Table 1:** Sociodemographic and socioeconomic characteristics of the latent classes of intersectional social status

	Indicators of intersectional social status						
	Class 1 (*n* = 100)	Class 2 ( *n* = 107)	Class 3 (*n*=106)	Class 4 (*n*=153)	Class 5 (*n*=100)	Class 6 (*n*=181)	Class 7 (*n*=305)
	*n*(%)	*n*(%)	*n*(%)	*n*(%)	*n*(%)	*n*(%)	*n*(%)
Ethnicity							
White British	0	103(97.2)	42(39.8)	74(46.5)	52(52.0)	0	265(86.0)
Black Caribbean	21(20.8)	0	8(7.9)	37(25.4)	8(8.5)	0	11(3.6)
Black African	30(29.8)	0	27(26.0)	9(5.5)	6(6.4)	62(33.8)	1(0.5)
White Other	24(24.3)	0	8(6.6)	11(7.7)	19(18.3)	71(38.0)	14(4.6)
Non-White Other	17(16.6)	0	12(10.9)	11(6.7)	13(12.7)	39(22.5)	6(2.3)
Mixed	8(8.5)	3(2.8)	9(8.8)	11(8.2)	2(2.1)	9(5.7)	8(3.0)
Migrant status							
Born in the UK	25(28.0)	107(100)	79(76.8)	110(74.9)	52(54.7)	10(6.8)	285(95.1)
Migrant (0-10)	11(12.1)	0	17(14.4)	6(4.3)	9(10.1)	81(47.3)	2(0.6)
Migrant (11-20)	27(28.5)	0	8(8.2)	16(11.1)	4(4.4)	52(28.6)	3(1.0)
Migrant (21+)	37(31.5)	0	1(0.7)	17(9.7)	31(30.8)	37(17.2)	10(3.3)
Educational attainment							
No qualifications/GCSE	47(45.0)	88(80.7)	13(12.1)	75(47.9)	19(17.6)	12(6.8)	6(1.8)
A Level	35(36.9)	17(17.4)	55(52.3)	65(43.4)	13(12.7)	45(24.4)	32(11.1)
Degree or above	18(18.1)	2(1.9)	38(35.6)	13(8.7)	68(69.7)	124(68.8)	267(87.1)
Social occupational class							
Class I	0	0	0	1(0.7)	0	23(13.3)	59(19.4)
Class II	0	0	0	23(14.6)	0	86(47.2)	197(64.4)
Class IIINM	0	0	0	44(27.8)	0	29(16.9)	30(10.2)
Class IIIM	0	0	0	27(18.7)	0	9(5.1)	16(4.9)
Class IV	0	0	0	46(30.3)	0	26(14.0)	3(1.0)
Class V	0	0	0	12(8.0)	0	7(3.5)	0
No SOC assigned	100(100)	107(100)	106(100)	0	100(100)	0	0
Employment status							
Full/part-time employed	0	0	0	153(100)	0	180(100)	305(100)
Student	6(8.0)	0	78(76.0)	0	0	0	0
Unemployed	25(29.1)	27(28.8)	28(24.0)	0	16(16.6)	0	0
Temporary sick/disabled	19(19.0)	18(19.2)	0	0	4(5.0)	0	0
Retired	30(24.2)	56(45.8)	0	0	53(50.2)	0	0
Looking after children	20(19.7)	6(6.2)	0	0	27(28.2)	0	0
Household income							
£0 - £12,096	59(68.6)	53(56.8)	14(17.0)	25(16.6)	11(11.7)	11(6.3)	3(0.8)
£12,097-£31,494	22(23.2)	41(43.2)	14(17.5)	71(51.1)	25(29.1)	38(21.5)	29(9.6)
£31495+	7(8.2)	0	49(65.5)	43(32.3)	49(59.2)	122(72.2)	261(89.6)
Any debt							
No	61(57.5)	86(76.7)	88(83.4)	104(69.1)	99(99.0)	164(90.3)	289(94.3)
Yes	39(42.5)	21(23.3)	18(16.6)	49(30.9)	1(1.0)	17(9.7)	16(5.7)
Any benefits							
No	20(16.4)	39(31.6)	87(83.8)	108(72.1)	87(86.5)	162(89.6)	294(96.6)
Yes	80(83.6)	68(68.4)	19(16.2)	45(27.9)	13(13.5)	19(10.4)	11(3.4)
Tenure							
Own outright/ mortgage	0	3(2.9)	31(30.7)	27(16.3)	88(89.4)	57(29.3)	199(66.4)
Private rented	18(19.9)	10(8.8)	32(29.9)	16(11.6)	5(6.2)	73(45.3)	68(26.9)
Social housing	79(80.1)	93(87.6)	17(15.2)	101(66.7)	4(4.4)	39(21.3)	15(4.6)
Other	0	1(0.8)	24(24.2)	7(5.4)	0	7(4.1)	6(2.1)
Moved in past 2 years							
Not moved or moved once	89(89.6)	106(98.8)	88(85.2)	145(94.6)	97(100)	157(85.7)	262(90.4)
Moved twice or more	9(10.4)	1(1.2)	16(14.8)	7(5.4)	0	20(14.3)	26(9.6)
Gender							
Male	32(35.5)	44(45.8)	48(52.3)	60(45.5)	32(35.2)	77(48.6)	144(53.6)
Female	68(64.5)	63(54.2)	58(47.7)	93(54.5)	68(64.8)	104(51.4)	161(46.4)
Age							
16-34	28(34.1)	13(16.6)	93(91.1)	52(44.0)	15(18.3)	67(44.3)	105(41.5)
35-54	35(35.5)	23(23.8)	13(8.9)	70(40.9)	22(22.2)	88(44.7)	155(47.5)
55+	37(30.4)	71(60.0)	0	31(15.1)	63(59.5)	26(11.0)	45(11.0)

*Latent classes;* (1) ‘Migrant, mixed ethnicity, low SES’ (n=100), (2) ‘White British, low SES’ (n=107), (3) ‘Non migrant, mixed ethnicity, student’ (n=106), (4) ‘Non migrant, mixed ethnicity, skilled’ (n=153), (5) ‘Mixed migration status, mixed ethnicity, economically inactive’ (n=100), (6) ‘Migrant, mixed ethnicity, high SES’ (n=181) and (7) ‘White British, high SES’ (n=305)

#### Statistical analysis

**Fig. 1:**
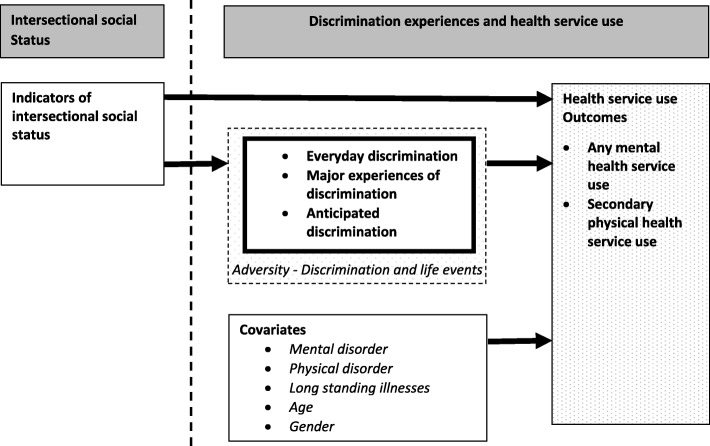
Model of intersectional social status, discrimination experiences and health service use

In order to explore associations between the key variables in this study as outline in Fig. 1, analyses were conducted in STATA 14.1 and survey commands were used to account for clustering by household and to generate standard robust errors (Statacorp, 2009). Weights were applied for within household non-response and sample attrition between SELCoH I and SELCoH II. Descriptive statistics were reported in terms of unweighted frequencies and weighted percentages. Non parametric tests (chi-square) explored associations between discrimination experiences, mental/physical disorder and long standing illness with intersectional social status in Table 2. Associations between intersectional social statuses with health service use outcomes were explored with the use of logistic regression models. Further analyses are presented for associations between single indicators of social status (ethnicity, migration status, educational attainment, social occupational class, employment status, household income, debt, benefit receipt, tenure and residential mobility) with health service use outcomes which were also explored with the use of logistic regression models. Odds ratios (ORs) with corresponding 95% confidence intervals (CI) were estimated in unadjusted models and models adjusting for age (continuous), gender, common mental disorder (continuous CIS-R score), physical disorder (continuous PHQ-15 score) and long-standing illness in Table 3 and Table 4. Associations between discrimination experiences with health service use outcomes were also explored with the use of logistic regression models. Odds ratios (ORs) with corresponding 95% confidence intervals (CI) were estimated in unadjusted models and two adjusted models in Table 5. The first model adjusted for all forms of discrimination simultaneously alongside intersectional social status, age (continuous), gender, common mental disorder (continuous score), physical disorder (continuous score) and long-standing illness. The second model made additional adjustments for life events.

**Table 2:** Prevalence of discrimination experiences and health status by intersectional social status

Stressors and health symptoms	Indicators of intersectional social status							
	Migrant, mixed ethnicity, low SES	White British, low SES	Non migrant, mixed ethnicity, student	Non migrant, mixed ethnicity, skilled	Mixed migration status, mixed ethnicity, economically inactive	Migrant, mixed ethnicity, high SES	White British, high SES	
	n (%)	*n (%)*	*n* (%)	*n (%)*	*n (%)*	*n (%)*	*n (%)*	*p*
Everyday discrimination								
Below median	41(36.9)	37(30.9)	28(26.2)	39(23.6)	49(48.1)	70(36.8)	153(49.6)	<0.001
Median or above	59(63.1)	70(69.1)	78(73.8)	114(76.4)	51(51.9)	111(63.2)	151(50.4)	
Reason for everyday discrimination								
Race/ethnicity	16(23.7)	1(1.4)	15(17.3)	30(26.4)	14(20.1)	51(36.3)	19(7.9)	<0.001
National origins	9(12.2)	0(0)	3(2.8)	1(0.8)	2(2.7)	12(7.4)	3(1.4)	
Education/income	3(4.2)	10(12.6)	4(3.7)	16(13.1)	4(5.2)	7(4.5)	22(10.1)	
Age	5(9.3)	13(16.4)	14(14.9)	10(7.8)	11(14.7)	7(5.5)	40(17.9)	
Gender	3(5.0)	0(0)	12(11.6)	4(2.8)	8(9.8)	9(5.8)	30(11.7)	
Other	27(45.7)	50(69.6)	44(49.7)	58(49.1)	31(47.4)	56(40.5)	119(51.1)	
Major discrimination								
0 domains	43(39.6)	51(43.4)	57(53.2)	72(45.5)	54(54.3)	83(45.9)	181(60.1)	<0.001
1-2 domains	28(29.6)	35(34.8)	31(30.4)	61(41.3)	39(39.1)	71(40.3)	97(31.8)	
3+ domains	29(30.8)	21(21.8)	17(16.4)	20(13.2)	6(6.7)	27(13.9)	27(8.1)	
Anticipated discrimination								
0 domains	57(55.4)	77(67.7)	70(68.4)	115(74.7)	79(79.3)	122(66.3)	236(77.1)	0.008
1 domain	30(30.6)	19(20.9)	28(24.7)	27(18.5)	17(17.7)	44(25.2)	56(18.6)	
2+ domains	13(14.0)	11(11.4)	8(7.0)	11(6.8)	3(3.0)	15(8.5)	13(4.3)	
Mental disorder	42(44.9)	42(41.1)	28(25.5)	33(20.6)	15(14.3)	30(16.2)	41(13.2)	<0.001
Physical disorder	25(28.9)	34(38.9)	19(17.6)	28(17.7)	9(9.5)	18(9.5)	24(7.6)	<0.001
Long standing illness	69(67.4)	88(80.4)	39(34.9)	70(40.2)	60(59.3)	67(35.2)	95(30.3)	<0.001

Weighted percentages to account for survey design; frequencies are unweighted and may not add up due to missing values

*P-*value for Pearson’s χ2 with Rao & Scott corrections for χ2 tests

Reason for discrimination – Other contains appearance, weight, sexual orientation, religion, physical disability and mental illness

**Table 3:** Prevalence estimates and odds ratios for associations between intersectional social status with health service use

	Health service use (last 12 months)					
	Any mental health service use			Secondary physical health service use		
	*n* (%)	OR 95% (CI)^a^	OR(95% CI) ^b^	*n* (%)	OR(95% CI) ^a^	OR(95% CI) ^b^
Total	181(17.1)			530(48.0)		
Indicators of intersectional social identity						
Migrant, mixed ethnicity, low SES	30(30.5)	4.29(2.37-7.74)	1.46(0.73-2.93)	53(49.8)	1.33(0.83-2.14)	0.59(0.36-0.99)
White British, low SES	21(20.5)	2.52(1.33-4.78)	0.88(0.40-1.90)	74(67.2)	2.74(1.67-4.50)	1.00(0.60-1.68)
Non migrant, mixed ethnicity, student	27(23.2)	2.95(1.63-5.33)	1.83(0.93-3.58)	52(47.7)	1.22(0.78-1.91)	1.48(0.88-2.49)
Non migrant, mixed ethnicity, skilled	26(16.1)	1.87(1.04-3.38)	1.27(0.65-2.47)	81(49.7)	1.32(0.89-1.98)	1.07(0.69-1.67)
Mixed migration status, mixed ethnicity, economically inactive	19(20.0)	2.44(1.28-4.64)	2.27(1.11-4.64)	61(61.3)	2.12(1.32-3.39)	1.16(0.66-2.03)
Migrant, mixed ethnicity, high SES	28(15.7)	1.81(1.02-3.22)	1.63(0.88-3.00)	72(37.8)	0.81(0.56-1.19)	0.72(0.47-1.09)
White British, high SES (reference group)	30(9.3)	1.00	1.00	137(42.8)	1.00	1.00

Weighted percentages to account for survey design; frequencies are unweighted and may not add up due to missing values

*OR* odds ratio, *CI* confidence interval

^a^ Unadjusted models

^b^ Adjusted for age (continuous variable), gender, mental/physical disorder and long standing illness

**Table 4:** Prevalence estimates and odds ratios for associations between single indicators of social status with health service use

	Health service use (last 12 months)					
	Any mental health service use (*n*=181)			Secondary physical health service use (*n*=530)		
	*n* (%)	Unadjusted OR (95% CI)	Adjusted OR^1^ (95% CI)	*n* (%)	Unadjusted OR (95% CI)	Adjusted OR^a^ (95% CI)
Ethnicity						
White British	75(14.1)	1.00	1.00	291(52.1)	1.00	1.00
Black Caribbean	15(15.4)	1.11(0.60-2.03)	1.08(0.53-2.21)	43(49.1)	0.89(0.55-1.44)	0.91(0.52-1.58)
Black African	20(14.2)	1.01(0.58-1.77)	0.85(0.43-1.67)	58(41.3)	0.65(0.44-0.96)	0.70(0.45-1.06)
White Other	47(33.9)	3.13(2.00-4.91)	2.86(1.74-4.68)	71(45.9)	0.78(0.53-1.14)	0.61(0.40-0.92)
Non White Other	17(17.0)	1.25(0.71-2.19)	1.04(0.55-1.95)	42(40.6)	0.63(0.40-0.99)	0.62(0.38-1.00)
Mixed	7(12.9)	0.91(0.39-2.11)	0.83(0.35-1.93)	25(44.5)	0.74(0.41-1.31)	0.83(0.45-1.55)
Migration status						
Born in the UK	106(15.8)	1.00	1.00	355(50.4)	1.00	1.00
Migrant (0-10)	21(16.4)	1.04(0.61-1.79)	1.21(0.67-2.17)	48(36.2)	0.56(0.38-0.83)	0.68(0.44-1.03)
Migrant (11-20)	25(22.6)	1.55(0.92-2.62)	1.52(0.84-2.75)	48(41.8)	0.71(0.47-1.07)	0.72(0.46-1.13)
Migrant (21+)	29(20.7)	1.39(0.86-2.25)	1.21(0.71-2.05)	76(52.7)	1.10(0.76-1.57)	0.60(0.40-0.92)
Educational attainment						
No qualifications/GCSE	45(17.8)	1.20(0.79-1.83)	0.60(0.35-1.04)	143(52.3)	1.41(1.04-1.91)	0.77(0.54-1.11)
A Level	54(20.0)	1.38(0.93-2.05)	0.89(0.57-1.39)	143(52.1)	1.39(1.02-1.90)	1.28(0.91-1.80)
Degree or above	82(15.3)	1.00	1.00	244(43.9)	1.00	1.00
Social occupational class						
Class I/II	48(11.7)	1.00	1.00	165(40.0)	1.00	1.00
Class III	19(12.2)	1.04(0.57-1.89)	0.88(0.47-1.67)	85(51.9)	1.62(1.09-2.41)	1.51(0.98-2.32)
Class IV/V	16(16.6)	1.50(0.80-2.80)	1.15(0.56-2.37)	40(41.2)	1.05(0.66-1.66)	0.91(0.56-1.47)
No SOC assigned	97(23.7)	2.34(1.57-4.47)	1.26(0.80-1.97)	240(55.5)	1.87(1.40-2.51)	1.26(0.90-1.76)
Employment status						
Full/part-time employed	83(12.5)	1.00	1.00	290(43.1)	1.00	1.00
Student	19(20.3)	1.78(0.99-3.21)	1.33(0.66-2.70)	40(46.3)	1.14(0.73-1.76)	1.44(0.84-2.47)
Unemployed	31(30.6)	3.08(1.88-5.04)	1.96(1.11-3.47)	46(45.9)	1.12(0.72-1.73)	0.87(0.55-1.43)
Temporary sick/disabled	22(53.0)	7.86(4.02-15.36)	1.74(0.74-4.10)	30(73.0)	3.58(1.74-7.35)	1.13(0.51-2.50)
Retired	16(12.1)	0.96(0.54-1.72)	0.53(0.25-1.12)	102(73.1)	3.59(2.35-5.50)	1.45(0.82-2.54)
Looking after children	9(18.0)	1.53(0.72-3.25)	1.05(0.46-2.43)	22(42.1)	0.96(0.54-1.71)	0.80(0.42-1.50)
Household income						
£0 - £12,096	40(23.4)	1.77(1.14-2.73)	0.85(0.49-1.46)	90(48.3)	1.13(0.80-1.61)	0.59(0.39-0.89)
£12,097-£31,494	44(17.7)	1.25(0.83-1.88)	0.90(0.56-1.45)	133(52.3)	1.33(0.96-1.84)	0.91(0.63-1.32)
£31495+	77(14.7)	1.00	1.00	250(45.1)	1.00	1.00
Any debt						
No	123(13.6)	1.00	1.00	452(48.0)	1.00	1.00
Yes	58(35.1)	3.44(2.37-5.00)	1.99(1.26-3.16)	78(47.9)	0.99(0.71-1.40)	0.80(0.53-1.21)
Any benefits						
No	106(13.3)	1.00	1.00	396(47.3)	1.00	1.00
Yes	75(29.0)	2.66(1.89-3.76)	1.39(0.91-2.11)	134(50.2)	1.12(0.85-1.48)	0.69(0.50-0.96)
Tenure						
Own outright/ mortgage	63(15.3)	1.00	1.00	208(49.5)	1.00	1.00
Rent/private	42(19.2)	1.32(0.84-2.05)	1.16(0.72-1.88)	91(39.0)	0.65(0.46-0.91)	0.82(0.56-1.20)
Rent/council	69(19.7)	1.35(0.92-2.00)	0.71(0.45-1.13)	195(53.5)	1.17(0.87-1.57)	0.90(0.65-1.24)
Other	5(11.5)	0.72(0.27-1.91)	0.48(0.13-1.76)	22(48.0)	0.94(0.50-1.76)	1.38(0.69-2.73)
Moved in past 2 years						
Not moved or moved once	162(17.1)	1.00	1.00	494(50.1)	1.00	1.00
Moved twice or more	17(21.3)	1.31(0.73-2.37)	1.63(0.85-3.10)	24(29.6)	0.42(0.26-0.69)	0.56(0.32-0.98)

*OR* odds ratio, *CI* confidence interval

^a^Adjusted for age (continuous variable), gender, mental/physical disorder and long standing illness

**Table 5:** Prevalence estimates and odds ratios for associations between discrimination experiences with health service use

	Health service use (last 12 months)							
	Health service use for mental health (n=181)				Secondary physical health service use (n=530)			
	*n* (%)	OR(95% CI)^a^	OR(95% CI)^b^	OR^b^ (95% CI)^c^	*n* (%)	OR(95% CI)^a^	OR(95% CI)^b^	OR(95% CI)^c^
Everyday discrimination								
Below median	52(12.5)	1.00			213(48.8)	1.00		
Median or above	129(19.9)	1.74(1.21-2.49)	1.28(0.85-1.93)	1.23(0.81-1.88)	317(47.6)	0.95(0.74-1.24)	0.84(0.62-1.16)	0.87(0.63-1.19)
Major discrimination								
0 domains	79(14.7)	1.00	1.00	1.00	255(44.6)	1.00	1.00	1.00
1-2 domains	59(15.6)	1.07(0.74-1.55)	0.71(0.46-1.10)	0.68(0.43-1.06)	181(47.8)	1.14(0.87-1.49)	1.12(0.82-1.53)	1.14(0.83-1.56)
3+ domains	43(29.4)	2.42(1.55-3.76)	0.84(0.47-1.50)	0.74(0.39-1.41)	92(59.9)	1.85(1.26-2.73)	1.38(0.87-2.18)	1.39(0.86-2.25)
Anticipated discrimination								
0 domains	106(13.6)	1.00	1.00	1.00	364(45.7)	1.00	1.00	1.00
1 domain	49(22.9)	1.89(1.28-2.80)	1.28(0.79-2.09)	1.36(0.83-2.22)	120(52.3)	1.30(0.95-1.79)	1.28(0.89-1.85)	1.28(0.88-1.85)
2+ domains	26(34.4)	3.34(1.92-5.82)	1.91(1.01-3.63)	1.85(0.93-3.71)	45(57.8)	1.63(0.98-2.70)	1.38(0.78-2.43)	1.29(0.72-2.31)
Indicators of intersectional social identity								
Migrant, mixed Ethnicity, low SES			1.38(0.68-2.81)	1.26(0.60-2.63)			0.54(0.32-0.93)	0.51(0.30-0.89)
White British, low SES			0.83(0.38-1.80)	0.81(0.37-1.77)			0.98(0.58-1.66)	1.03(0.60-1.75)
Non migrant, mixed ethnicity, student			1.72(0.87-3.40)	1.85(0.93-3.68)			1.45(0.86-2.46)	1.47(0.86-2.51)
Non migrant, mixed ethnicity, skilled			1.22(0.63-2.36)	1.21(0.63-2.34)			1.08(0.69-1.69)	1.06(0.68-1.67)
Mixed migration status, mixed ethnicity, economically inactive			2.28(1.11-4.69)	2.32(1.11-4.84)			1.15(0.65-2.03)	1.18(0.67-2.09)
Migrant, mixed ethnicity, high SES			1.56(0.84-2.90)	1.57(0.84-2.95)			0.69(0.45-1.03)	0.71(0.46-1.07)
White British, high SES (reference group)			1.00	1.00			1.00	1.00

*OR* odds ratio, *CI* confidence interval

^a^Unadjusted models

^b^Adjusted for intersectional social status, age (continuous variable), gender, mental/physical disorder, long standing illness and discrimination experiences

^c^As model 2 with additional adjustments for life events

## Results

### Characteristics of the sample

Approximately 46% of the sample were assigned to one of the two latent classes characterized by high SES; the ‘White British, high SES’ (29%) and ‘Migrant, mixed ethnicity, high SES’ (17%) classes (Table 6). The remaining five latent classes comprised of two low SES classes, a student class, a skilled worker class and an economically inactive class with relatively high SES that all varied by migration status and ethnicity. The ‘White British, high SES’ class represents the most privileged intersectional social status while the ‘Migrant, mixed ethnicity, low SES’ class represents the most disadvantaged. The other five latent classes represent intersectional social statuses characterized by both privilege and disadvantage in various ways (see Table 1). The majority of the sample experienced some form of everyday discrimination (62%). Reported reasons for these experiences included race/ethnicity (18.7%), age (12.7%) and education/income (8.0%), while 49.6% of the sample attributed these experiences to ‘other’ reasons (e.g. general appearance or other). Approximately 14% of the sample reported experiencing major discrimination in three or more life domains and almost a third reported anticipated discrimination in at least 1 domain. 48% of the sample had used secondary physical health services and approximately 17% of the sample had used health services in relation to mental disorder in the past year.

**Table 6:** Sample characteristics

	Total sample
	*n* (%)
Total	1052
Indicators of intersectional social status	
Class 1: Migrant, mixed ethnicity, low SES	100(9.5)
Class 2: White British, low SES	107(9.3)
Class 3: Non migrant, mixed ethnicity, student	106(12.9)
Class 4: Non migrant, mixed ethnicity, skilled	153(14.3)
Class 5: Mixed migration status, mixed ethnicity, economically inactive	100(8.2)
Class 6: Migrant, mixed ethnicity, high SES	181(17.1)
Class 7: White British, high SES	305(28.7)
Age	
17-39	479(54.0)
40-59	381(31.7)
60+	192(14.3)
Gender	
Female	615(52.5)
Male	437(47.5)
Everyday discrimination	
Below median	417(37.6)
Median or more	634(62.4)
Reason for everyday discrimination	
Race/ethnicity	146(18.7)
National origins	30(3.4)
Education/income	66(8.0)
Age	100(12.7)
Gender	66(7.6)
Other	385(49.6)
Major discrimination	
0 domains	541(50.7)
1-2 domains	362(35.1)
3+ domains	147(14.2)
Anticipated discrimination	
0 domains	756(71.0)
1 domain	221(21.8)
2+ domains	74(7.2)
Any mental health service use	181(17.1)
Secondary physical health service use	530(48.0)
Mental disorder	231(22.1)
Physical disorder	157(15.4)
Long standing illness	488(43.9)
Life events	
0-3 events	464(46.4)
4-7 events	435(43.5)
8+ events	104(10.1)

Weighted percentages to account for survey design; frequencies are unweighted and may not add up due to missing values.

Reason for discrimination – Other contains appearance, weight, sexual orientation, religion, physical disability and mental illness

### Discrimination and health across intersectional social status groups

There were notable differences in the distribution of discrimination experiences and health status by intersectional social status (Table 2). Those in the ‘White British, high SES’ class and the ‘Mixed migration status, mixed ethnicity, economically inactive’ class had the lowest proportions of reporting everyday discrimination, any major discrimination and any anticipated discrimination. In contrast, the two latent classes characterized by low SES had higher proportions of reporting any major discrimination and any anticipated discrimination compared to the other latent classes. Notably, approximately 45% of the ‘Migrant, mixed ethnicity, low SES’ class reported anticipated discrimination in at least one domain. There were also differences in the perceived reason for experiencing discrimination in everyday life across the classes. For example, while the two migrant classes more commonly perceived these experiences were due to their ethnicity and migration status, the ‘White British, low SES’ class more commonly attributed these experiences to education/income and age whilst the ‘White British, high SES’ class more commonly attributed these experiences to age and gender. In terms of health status, the ‘Migrant, mixed ethnicity, low SES’ and ‘White British, low SES’ classes had the highest prevalence of both mental and physical disorder. Over 40% of the members in each of these two classes met the criteria for CMD. In contrast, the ‘White British, high SES’ class had the lowest prevalence of both mental and physical disorder, at 13.2 and 7.6% respectively. The expected gradient from most advantaged to most disadvantaged intersectional social status is observed for the prevalence of both mental and physical disorder. However, the prevalence of long standing illness in the ‘Mixed migration status, mixed ethnicity, economically inactive’ is higher than expected based on their level of relative financial and educational advantage.

### Health service use by intersectional social status groups

#### Health service use for mental disorder

In unadjusted logistic regression models, all latent classes had increased odds of reporting health service use in relation to mental disorder compared to the ‘White British, high SES’ reference class (Table 3). Notably, the most disadvantaged ‘Migrant, mixed ethnicity, low SES’ class was associated with four times higher odds of reporting health service use in relation to mental disorder in comparison to the reference class. After adjustments for age, gender and health status, only the ‘Mixed migration status, mixed ethnicity, economically inactive’ classes remained associated with increased health service use for mental disorder (OR: 2.27; 95% CI: 1.11–4.64), despite only 14.3% of this class reported symptoms of mental disorder. These findings did not support our first hypothesis that those with multiple disadvantaged social statuses would have decreased health service use. In comparison to the findings using single indicators of social status (presented in Table 4), the differences identified using an intersectional approach were notably different. White Other ethnicity was associated with increased health service use for mental disorder in models adjusting for age, gender and health status (OR: 2.86; 95% CI: 1.74–4.68). However, the latent class of social status with the highest proportion of those who identified as White Other (38%), the ‘Migrant, mixed ethnicity, high SES’ class, was not associated with increased health service use for mental disorder. Unemployment and debt were also associated with increased health service use for mental disorder. Despite high levels of debt and unemployment in the ‘Migrant, mixed ethnicity, low SES’ and ‘White British, low SES’ classes (Table 1), these classes were not associated with increased health service use for mental disorder.

#### Health service use for physical disorder

Consistent with the above findings for mental disorder, the ‘White British, low SES’ and ‘Mixed migration status, mixed ethnicity, economically inactive’ classes also had increased odds of reporting secondary physical health service use in unadjusted models, relative to the ‘White British, high SES’ reference group (Table 3). However, after adjusting for age, gender and health status these associations were fully attenuated. Notably, the ‘Migrant, mixed ethnicity, low SES’ class was associated with decreased secondary physical health service use in this model (OR: 0.59; 95% CI: 0.36–0.99), despite being one of the groups with the poorest health status. This partially supports our hypothesis that those with multiple disadvantaged social statuses would use less health services than those with privileged or singularly disadvantaged social status. Similarly, being a long-term migrant (residing in the UK for 21 years or more), White Other ethnicity, low household income, benefit receipt and high residential mobility were all associated with decreased secondary physical health service use in adjusted models using single indicators of social status (Table 4).

#### Discrimination experiences and health service use

In unadjusted logistic regression models, all forms of discrimination were associated with increased odds of reporting health service use for mental disorder (Table 5). In particular, anticipating discrimination in two or more life domains were associated with over three times the odds of reporting health service use for mental disorder (OR: 3.34; 95% CI: 1.92–5.82). After adjusting for all discrimination experiences simultaneously alongside intersectional social status, age, gender and health status, only anticipated discrimination remained associated with increased health service use for mental disorder. In terms of secondary physical health service use, no associations between discrimination and health service use were significant except that reporting major experiences of discrimination within three or more life domains was associated with increased health service use in unadjusted models. However, this association was fully attenuated after adjusting for other types of discrimination experiences simultaneously alongside intersectional social status, age, gender and health status. Further controlling for discrimination experiences and life events did appear to have a small attenuating effect for the association between the ‘Migrant, mixed ethnicity, low SES’ class and health service use for both mental and physical disorder. However, these attenuations were not in the expected direction. Thus, there was no support for our hypothesis that discrimination experiences would act as a barrier to health service use.

## Discussion

### Main findings

Findings from this study illustrate the importance of taking an intersectional approach for health service use research. Using latent class analysis allowed us to identify a class characterized by multiple disadvantages that was associated with decreased secondary physical health service use after controlling for age, gender and health status. This partially supported our hypothesis that those with multiple disadvantaged social statuses would have decreased health service use in comparison to those with singular disadvantaged or privileged social status. However, contrary to our hypothesis, we also identified a ‘Mixed migration status, mixed ethnicity, economically inactive’ class, characterized by both disadvantage and financial/educational privilege, to be associated with increased health service use for mental disorder. We further hypothesized that discrimination experiences would act as a barrier to health service use. However, this hypothesis was not supported. In fact, some aspects of discrimination appeared to increase rather than decrease the odds of health service use; anticipated discrimination was associated with twice the odds of reporting health service use for mental disorder in adjusted models. Associations between other forms of discrimination and health service use were fully attenuated. These attenuations were predominantly driven by controlling for health status (data not shown), suggesting that discrimination experiences may result in more health service need by increasing the risk of mental and physical disorder e.g. associations are driven by increased need in these groups.

### Discrimination and health service use

Findings from the current study related to the role of discrimination experiences in health service use address the paucity of research in this area and the limited focus in the literature on racial discrimination as a barrier to health service use [10, 11]. Contrary to previous studies, our findings seem to indicate that discrimination experiences across multiple life domains act as stressors that contribute to help seeking due to increased mental disorder for those experiencing multiple disadvantage [38]. However, previous studies that suggest discrimination acts as a barrier to engagement with health services are predominantly based in the United States and therefore findings may be specific to the healthcare context of that country [6]. It is also possible that the presence of a mental disorder is associated with increased anticipated discrimination through elevated general tendencies for negative expectations often associated with such problems. However, controlling for prior mental disorder (mental disorder at SELCoH 1) did not attenuate associations between anticipated discrimination and health service use (data not shown). Discrimination was most prevalent in the ‘Migrant, mixed ethnicity, low SES’ class, and its impact on the association between this class and health service use suggest that it may have more pronounced effect on multiple disadvantaged groups. This builds on previous findings on the differential impact of discrimination on CMD by ethnicity and migration status [4] and the manifestation of hypervigilance for such groups in contexts where they feel more vulnerable [39]. There was no evidence in the current study to suggest that discrimination experiences were acting as a barrier to health service use, consistent with a growing number of studies finding few or no relationships with service use [40]. As a recent systematic review suggested, discrimination experiences may have more important implications for patient experience, quality of healthcare provision and delaying service use rather than rates of service utilization [6]. Future studies would benefit from capturing multiple forms of discrimination across life domains and examining the mechanisms through which discrimination may both increase the need for and act as a barrier to health service use.

### Intersectional approaches to health service use research

While results from conventional analyses using single indicators of social status replicated previous findings related to inequalities in secondary health service use, utilizing intersectional analytic approaches highlighted the complex nature in which social statuses are simultaneously experienced through systems of inequality, processes of discrimination and access to healthcare [18, 19, 41]. The most disadvantaged class, characterized by being migrant, ethnic minority and of low SES, was associated with decreased secondary physical health service use. Despite a similar SES profile, being in the ‘White British, low SES’ class, was not associated with decreased secondary health service use. In fact, there were no differences observed between this class and the ‘White British, high SES’ reference class in terms of secondary physical health service use. Similarly, despite White Other ethnicity being associated with decreased secondary health service use, results from intersectional analyses suggested that associations differ for those who identify as White Other ethnicity at the intersection of SES, e.g. only those with low SES were associated with decreased secondary health service use. These results pertain to the simultaneous experience of multiple disadvantage at the intersection of migration status, ethnicity and SES as an important factor for understanding inequalities in health service use, broadly supported by the tenets of intersectionality theory [23]. Our results are also consistent with previous research that indicates that those with multiple disadvantaged status experience more discrimination and have poorer health than those with privileged or singularly disadvantaged social status [24, 25, 42–44]. The attribution for discrimination experiences also varied across intersectional social status classes. Understanding such variations in perceived reason for discrimination at these intersections of social status is likely to be important for tackling mistrust of health services and differences in service use.

Interestingly, the following single indicators of social status were associated with increased health service use for mental disorder; White Other ethnicity, being a long-term migrant (residing in the UK for 21 years or more), low household income and benefit receipt. Given that the majority of mental health service use in this sample was in primary care, this is consistent with previous literature on health service use in England which also found an association between low social status and increased use of primary care services after controlling comprehensively for health need [19]. In contrast, only one intersectional class, the ‘Mixed migration status, mixed ethnicity, economically inactive’ class was associated with increased health service use for mental disorder. Although this class was predominantly economically inactive, it was also characterized by high household income and high educational attainment. This finding could be linked to previous research on mental health literacy which highlights associations between higher educational attainment and increased help-seeking [45, 46]. In addition, this group’s economic inactivity may result in greater availability for accessing mental health services (which are typically provided within working hours). This highlights the importance of comparing results from both conventional and intersectional approaches, as well as the need to conduct further research to understand health service access at the intersection of ethnicity, migration status and SES.

### Strengths and limitations

The study analyses data from a large representative community study with a diverse sample of migrants and ethnic minorities. Seventy-three per cent of the sample was retained in SELCoH 2, with sample attrition more likely in participants who were younger, male and unemployed, but also among those who met the criteria for a CMD [4]. The diverse sample allowed examination of health service use inequalities using intersectional approaches to uncover previously unidentified risk groups that would not have been observed using single indicators of social status alone. However, latent classes are specific to the sample population and results may not be generalizable to other samples or urban contexts. Similarly, these results may not be directly comparable with studies in other countries that have differing health systems or do not provide universal access to healthcare. Other limitations related to sample size should be noted. We were unable to disaggregate health service use for mental disorder into primary and secondary services due to small cell sizes. Therefore, we were unable to focus on inequalities in secondary mental health care, where barriers to mental health service use for low status groups have been previously identified [41, 47]. There were also insufficient numbers to investigate discrimination experiences in healthcare separately. Moreover, we we were only able to focus on any health service use over a 12-month period and did not have detailed information on frequency of visits or quality of treatment. At the same time, there are further strengths to the study. Previous studies have shown that how we measure need-related factors at both the individual (e.g. health status) and contextual level (e.g. stressful life events) influence the conclusions we reach about inequalities in health service use, yet measuring multiple need-related factors within the same study is rare [18, 19]. The current study was able to measure current health symptoms, long standing illnesses, multiple levels of discrimination experiences (everyday, major and anticipated) and life events simultaneously. Such comprehensive data allowed us to not only examine health service use inequalities while better accounting for health status but also enabled us to investigate if discrimination experiences were acting as a barrier or stressor.

## Conclusion

This study highlights the complex ways in which discrimination experiences may increase the need for health services and differences in health service use at the intersection of ethnicity, migration status and SES. Future research should focus on how differing levels of discrimination (e.g. everyday, major and anticipated) interrelate to influence health and subsequent engagement with health services longitudinally, to understand how inequalities may be generated and perpetuated, particularly among those with multiple disadvantaged statuses. Exploring the processes that affect help-seeking or impact on quality of care may not only have important implications for clinical practice but also for addressing inequities.

## Abbreviations

CI: Confidence Interval
CIS-R: Clinical Interview Schedule Revised
CMD: Common Mental Disorder
GP: General Practitioner
LCA: Latent Class Analysis
OR: Odds Ratio
PHQ: Physical Health Questionnaire
SELCoH: South East London Community Health Study
SES: Socioeconomic Status
SOC: Social Occupational Class
